# Use of Latent Class Analysis and k-Means Clustering to Identify Complex Patient Profiles

**DOI:** 10.1001/jamanetworkopen.2020.29068

**Published:** 2020-12-11

**Authors:** Richard W. Grant, Jodi McCloskey, Meghan Hatfield, Connie Uratsu, James D. Ralston, Elizabeth Bayliss, Chris J. Kennedy

**Affiliations:** 1 Division of Research, Kaiser Permanente Northern California, Oakland; 2 Kaiser Permanente Washington Health Research Institute, Kaiser Permanente Washington, Seattle; 3 Institute for Health Research, Kaiser Permanente Colorado, Aurora; 4 Division of Epidemiology and Biostatistics, University of California, Berkeley, Berkeley

## Abstract

**Importance:**

Medically complex patients are a heterogeneous group that contribute to a substantial proportion of health care costs. Coordinated efforts to improve care and reduce costs for this patient population have had limited success to date.

**Objective:**

To define distinct patient clinical profiles among the most medically complex patients through clinical interpretation of analytically derived patient clusters.

**Design, Setting, and Participants:**

This cohort study analyzed the most medically complex patients within Kaiser Permanente Northern California, a large integrated health care delivery system, based on comorbidity score, prior emergency department admissions, and predicted likelihood of hospitalization, from July 18, 2018, to July 15, 2019. From a starting point of over 5000 clinical variables, we used both clinical judgment and analytic methods to reduce to the 97 most informative covariates. Patients were then grouped using 2 methods (latent class analysis, generalized low-rank models, with k-means clustering). Results were interpreted by a panel of clinical stakeholders to define clinically meaningful patient profiles.

**Main Outcomes and Measures:**

Complex patient profiles, 1-year health care utilization, and mortality outcomes by profile.

**Results:**

The analysis included 104 869 individuals representing 3.3% of the adult population (mean [SD] age, 70.7 [14.5] years; 52.4% women; 39% non-White race/ethnicity). Latent class analysis resulted in a 7-class solution. Stakeholders defined the following complex patient profiles (prevalence): high acuity (9.4%), older patients with cardiovascular complications (15.9%), frail elderly (12.5%), pain management (12.3%), psychiatric illness (12.0%), cancer treatment (7.6%), and less engaged (27%). Patients in these groups had significantly different 1-year mortality rates (ranging from 3.0% for psychiatric illness profile to 23.4% for frail elderly profile; risk ratio, 7.9 [95% CI, 7.1-8.8], *P* < .001). Repeating the analysis using k-means clustering resulted in qualitatively similar groupings. Each clinical profile suggested a distinct collaborative care strategy to optimize management.

**Conclusions and Relevance:**

The findings suggest that highly medically complex patient populations may be categorized into distinct patient profiles that are amenable to varying strategies for resource allocation and coordinated care interventions.

## Key Points

**Question:**

What distinct clinical profiles can be identified within a population of the most medically complex patients?

**Findings:**

In this cohort study of 104 869 individuals, data clustering methods were combined with clinical stakeholder assessment to define clinical profiles within the top 3% most medically complex adult patients in a large integrated care system: high acuity, older with cardiovascular complications, frail elderly, pain management, psychiatric illness, cancer treatment, and less engaged. These profiles had significantly different 1-year health care utilization and mortality, and each profile suggested different adjunctive care strategies.

**Meaning:**

The findings suggest that a single care model may not meet the needs of adults with high comorbidity and care utilization.

## Teaser

This cohort study uses data clustering methods and clinical stakeholder assessment to identify clinical profiles in a population of medically complex patients.

## Introduction

A small number of patients consume a large proportion of the total national health care budget.^1^ These medically and socially complex patients have been the target of efforts by health systems and insurers to bend the cost curve of increasing health care expenditures. To date, care management programs designed to address needs and reduce hospitalizations or disease progression in medically complex patients have shown only modest success.^2,3,4,5,6,7,8^ Recent randomized clinical trials^9,10^ have now shown substantial clinical benefit or cost savings despite the intuitive value of care management efforts, such as social work consultation, electronic registries, pharmacist consultations, home visits, and other care management strategies.

One recognized limitation of current care programs involves the initial step of patient identification.^11^ Individuals with complex medical and social care needs have traditionally been identified by prior year costs or care utilization, number and type of concurrent comorbid conditions, and/or predicted future hospitalization or costs. These approaches lack specificity and may contribute to the poor results seen for most prior care management interventions. Ideally, population-based care management programs for medically complex patients that include patient surveillance, tracking, and outreach by nurses, social workers, and other health care workers would be tailored to the different needs of distinct patient subgroups.^12^ This strategy would allow care resources to be more effectively allocated to address care barriers faced by different patients with otherwise similarly high levels of comorbidity, prior utilization, and predicted risk. There are limited empirical data to guide which patient subgroups exist within the overall medically complex patient population.

We sought to characterize clinical heterogeneity among patients with the highest medical complexity (defined by commonly used thresholds for comorbidity, health care utilization, and predicted hospitalization risk) within a large, integrated care delivery system using available electronic health record data. We tested the hypothesis that a data-driven approach could yield distinct, clinically meaningful patient profiles within this narrowly defined stratum of medically complex patients. Our overarching goals were to provide an empirical basis for conceptual models of patient medical complexity^13^ and to inform strategies for tailoring care for the most medically complex patients within a care system or network.

## Methods

### Setting and Participants

This cohort study was conducted within Kaiser Permanente Northern California (KPNC), an integrated care delivery system with 4.2 million members. KPNC provides care to a population insured through employer-based plans, Medicare, Medicaid, and the California health insurance exchange. Members are highly representative of the local populations.^14^ KPNC uses a single electronic health record for all inpatient and outpatient care, including all pharmacy orders and prescriptions dispensed. Any out-of-network care is also recorded through the KPNC external billing system. The Kaiser Permanente Institutional Review Board approved the study and granted permission for a waiver of consent for study participants as allowed under the Common Rule. This report followed the Strengthening the Reporting of Observational Studies in Epidemiology (STROBE) reporting guideline.^15^

Among the 3.3 million adult members of KPNC who were 18 years or older, we defined a cohort representing a narrow band of the most medically complex patients within the care system. Using a snapshot of data from July 15, 2018, we identified patients based on high comorbidity (Comorbidity Point Score, version 2 [COPS-2] >14)^16^ and high health care utilization (defined as a likelihood of hospitalization [LOH] score >0.25 and/or ≥2 emergency department [ED] admissions in the prior year).^17^ The COPS-2 is a validated, continuous comorbidity score based on hierarchical condition categories that predicts mortality; scores range from 0 to a theoretical maximum of 701, with higher scores indicating more comorbidities. The LOH score uses patient historical clinical data and logistic regression to predict the likelihood of admission in the next 6 months; scores range from 0 to 1, with a higher score indicating greater likelihood of admission. Because both these scores are highly skewed (with most adults having low scores), thresholds (14 for COPS-2, >0.25 for LOH) are used in clinical practice to identify patients with multiple chronic comorbidities and increased hospitalization risk. On the basis of the goals of care management programs for medically complex patients, we also included the 2 or more ED admissions in the prior year as an indicator of potentially preventable high cost care. We then linked all available electronic data for these study patients from the preceding 12 months for our primary analyses and for the subsequent 12 months (to July 15, 2019) for our outcome assessments.

### Analytic Process

Our goal was to define distinct patient clinical profiles through application of 2 independent, unsupervised grouping methods: latent class analysis (LCA) and k-means clustering with preprocessing by generalized low-rank models (GLRM).^18^ We implemented the following 4 steps to achieve this goal.

#### Merging and Formatting of Data

Data from the electronic health record included patient demographic information, medication prescriptions, health care utilization (outpatient, inpatient, and via secure online messages), medical diagnoses, patient-reported data (including self-reported exercise, alcohol use, and depression symptoms), vital signs, membership benefits, procedures, laboratory results, and orders for durable medical equipment (eg, wheelchair, home oxygen) (eTable 1 in the Supplement). Patient addresses were linked to census tracts to obtain the neighborhood deprivation index.^19^

#### Clinically Guided Variable Selection and Reduction

From the more than 5000 potential data elements available within the electronic health record identified in the first step, we created a more limited analytic data set by combining similar variables (eg, using medication group category to combine different types of benzodiazepines), selecting variables known to be important for gauging health status (eg, markers of frailty such as requiring a wheelchair), and excluding variables that were rare (<1% of cohort) or not informative (eg, common, benign dermatologic procedures). Decisions about variables were made by research team consensus (all authors). This clinically guided process also included assessment of statistical correlations using the Jaccard similarity metric^20^ and divisive clustering techniques (proc varclus in SAS, version 9.4 [SAS Institute]) to combine or remove redundant and highly correlated variables, thereby increasing our ability to extract meaningful information from a smaller set of key variables. Variables were dichotomized using common clinical thresholds (eg, abnormal laboratory result thresholds) and top quartile (eg, for count variables). This process resulted in a final set of 97 informative variables for our cluster analysis (eTable 2 in the Supplement). We excluded basic demographic variables (age, sex, and race/ethnicity) and cohort-defining variables (COPS-2, LOH score, and prior 12-month ED admissions) from the variable reduction and grouping process. These excluded variables were subsequently used to further describe the resulting data clusters.

#### Identification of Patient Clusters

We implemented 2 independent analytic strategies to identify distinct patient clusters within our narrowly defined band of medical complexity. We first used LCA, a model-based approach that defines patients by shared underlying unobserved characteristics. Latent class analysis is an iterative, maximum likelihood method that estimates how patterns in patient characteristics can be summarized into a finite number of groups, or latent classes, by providing a probability distribution over the cluster assignment for each patient. To investigate the extent to which these groupings remained stable across 2 methods, we separately applied k-means clustering, a non–model-based method that applies optimization algorithms to define patient clusters. This cluster assignment method is based on the minimum distance of a patient from the centroid of the cluster (ie, the sum of the deviation of each variable compared with the centroid values).

#### Interpretation of Cluster Results

We used a 3-fold strategy to derive clinical meaning from the clustering analysis results. First, we examined 1-year follow-up outcomes (ED admission, in-patient hospitalization, and mortality) by cluster. Second, we shared our analytic results with clinical stakeholders to define clinically meaningful complex patient profiles and suggest potential care strategies tailored to these distinct patient profiles. Third, we compared the patient groupings independently derived from the 2 parallel methods (LCA and k-means clustering) to investigate patterns of commonality and dissimilarity.

The clinical stakeholders were members of the KPNC Complex Needs Advisory Council, an interdisciplinary committee of 25 clinicians (hospital-based physicians, geriatricians, primary care physicians, pharmacy leaders, nurse care coordinators, and social workers) and clinical operational leaders. Membership on the advisory council was based on expertise and commitment to improving care for patients with complex medical and social needs. To define clinically meaningful complex patient profiles, stakeholders first formed small groups of 2 to 3 individuals to review the data during a 2-hour session; then each group presented to the overall committee, and consensus was reached on the patient profiles represented by each data cluster. These profiles reflected the group’s clinical experiences with different types of medically complex patients who they had cared for in their practices.

### Statistical Analysis

#### Latent Class Analysis

Latent class analysis is a finite mixture modeling method that assumes the overall population heterogeneity with respect to a distribution of observable response (ie, manifest) variables is the result of at least 2 or more unobserved, homogenous subgroups, known as latent classes. The scientific goal of LCA-based clustering was to arrive at a solution that represented the most parsimonious and interpretable set of classes. In this study, we selected the model with the fewest number of classes while ensuring both statistical and clinical significance. Latent class analysis was implemented using the poLCA package in R, version 1.1 (R Project for Statistical Computing). Class solution was based on examining bayesian information criterion, classification accuracy (mean posterior probability and the odds of correct classification within each class), and class homogeneity and separation (model-estimated prevalence) across 2 to 10 clusters.

#### GLRM Reduction and k-Means Clustering

We first transformed our data set of binary variables into continuous latent variables using GLRM, with settings selected through a grid search with 5-fold cross-validation to minimize reconstruction error. We then applied k-means clustering with the Euclidean distance metric, a non–model-based method not grounded by an underlying statistical model and typically corresponding to discrete optimization algorithms to optimize a diverse range of objective criteria. Optimal cluster number solution was determined by finding the highest mean Rand index obtained by running k-means clustering on 100 bootstrapped samples across 2 to 10 clusters.

## Results

### Patient Population

We identified 104 869 adults (3.3% of the adult population in KPNC) with a COPS-2 greater than 14 and either an LOH score greater than 25% or more than 2 ED admissions in the prior year; 86% had a low comorbidity score, 11% had a high comorbidity score only, and 3% had high comorbidity and utilization scores. The mean (SD) age of the sample was 70.7 (14.5) years, 52.4% were women, 39% were non-White race/ethnicity, and the mean (SD) COPS-2 was 72.7 (42.6). Patients with a COPS-2 of 72 were in the top 1 percentile of scores for the adult KPNC population, with multiple chronic conditions and a 1-year mortality of 13% (calculated in the population based on 877 patients with a COPS-2 of 72).

### LCA Results

**Figure 1.**
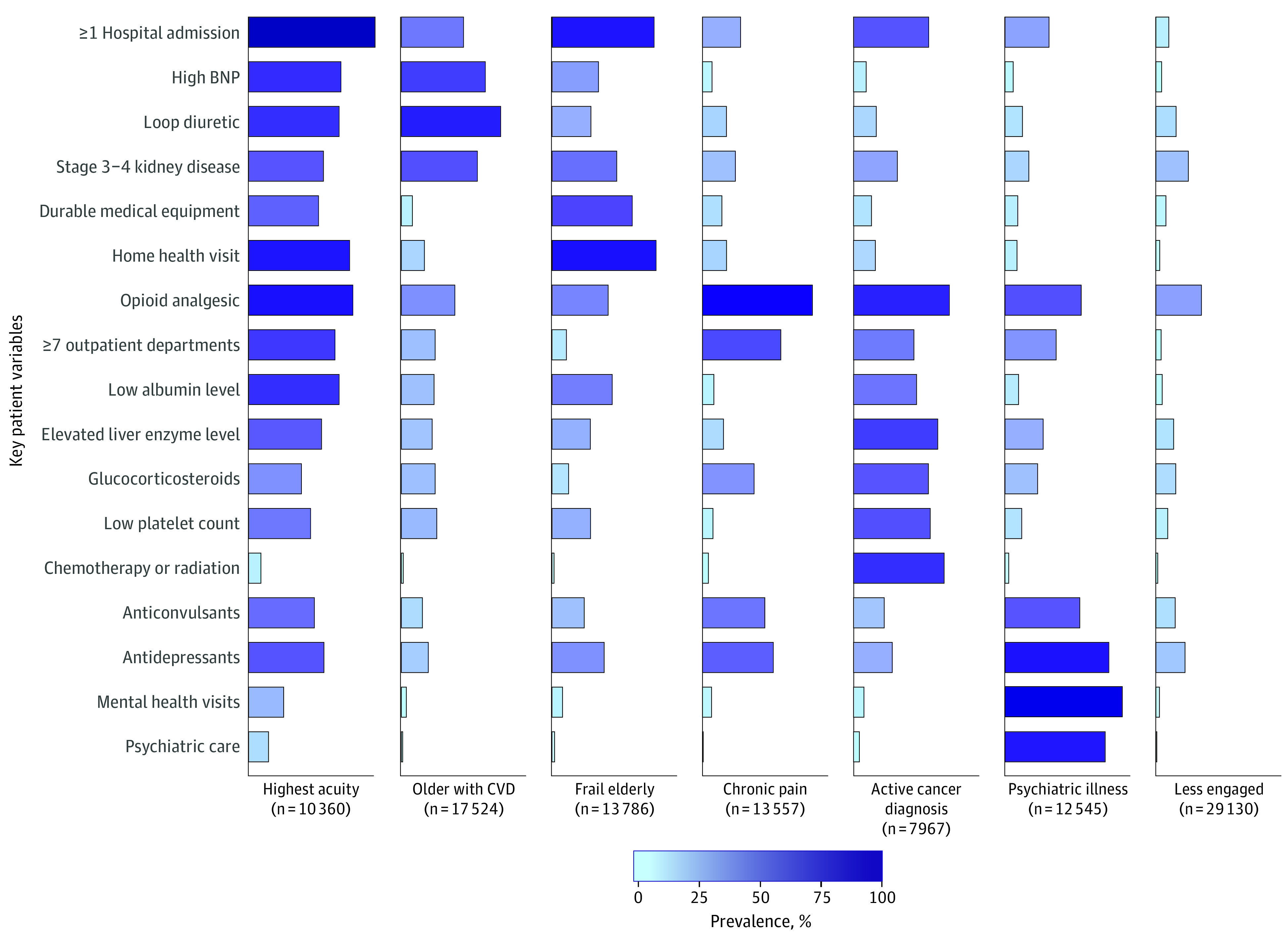
Seven Patient Profiles Derived From Latent Class Analysis The 17 variables were chosen from the 97 used in the latent class analysis model because they had the largest variation in prevalence across the 7 classes. Each listed variable had at least 55% prevalence in 1 or more class and less than 10% in other classes. BNP indicates brain natriuretic peptide; CVD, cardiovascular disease.

The optimal LCA solution yielded 7 patient groups. From the initial set of 97 variables used to define the groups, we identified the variables that had the largest variation in model-estimated prevalence across the 7 clusters. Figure 1 shows the 17 variables with at least 55% prevalence in 1 or more classes and less than 20% in other classes (details given in eTable 2 in the Supplement).^21^ The 7 groups had significant differences in 1-year outcomes, with mortality ranging from 3.0% to 23.4%, hospitalization from 18.3% to 51.2%, hospice admission from 1.6% to 17%, and mental health care visit from 5.3% to 59.8% (Table 1 and eTable 3 in the Supplement).

**Table 1.** Baseline and 1-Year Follow-up Characteristics of the Overall Population and by Patient Profile

Characteristic	Patients^a^							
	Total	Highest acuity	Older with CVD	Frail elderly	Pain management	Cancer treatment	Psychiatric illness	Less engaged
No. (%)	104 869 (100)	10 360 (9.9)	17 524 (16.7)	13 786 (13.1)	13 557 (12.9)	7967 (7.6)	12 545 (11.9)	29 130 (27.7)
Baseline								
Age, mean (SD), y	70.7 (14.5)	72.0 (12.1)	74.8 (11.5)	80.6 (11.4)	68.9 (13.0)	65.7 (12.7)	58.3 (14.6)	70.7 (14.7)
Women	52.4	51.8	40.7	57.8	60.0	49.8	61.4	50.3
Race/ethnicity								
Asian	10.4	9.2	11.9	10.5	6.4	15.0	4.8	12.9
Black	11.0	11.9	9.9	9.8	10.5	8.9	11.4	12.5
Hispanic	15.5	16.7	13.4	12.5	14.9	15.7	16.4	17.8
White	56.4	55.3	58.5	60.3	61.4	59.5	60.0	50.1
COPS-2, mean (SD)	73 (43)	124 (48)	82 (32)	93 (42)	56 (32)	90 (38)	50 (33)	52 (30)
LOH score, mean (SD)	0.4 (0.2)	0.7 (0.2)	0.4 (0.2)	0.5 (0.2)	0.3 (0.2)	0.5 (0.2)	0.3 (0.2)	0.3 (0.2)
Prior year ED visits, mean (SD)	1.9 (2.3)	2.6 (3.2)	1.3 (1.5)	1.6 (1.9)	2.2 (1.9)	1.3 (1.6)	2.9 (3.6)	1.7 (1.5)
Medication count, mean (SD)	5.0 (3.5)	7.2 (4.0)	6.4 (3.1)	4.0 (3.1)	5.6 (3.5)	4.0 (3.1)	5.0 (3.8)	3.7 (2.9)
1-y Follow-up								
Died	10.9	20.5	9.9	23.4	3.7	21.7	3.0	6.0
Admitted								
Hospice	6.4	10.2	3.7	17.0	2.0	15.5	1.6	3.2
Hospital	28.6	51.2	33.3	34.7	22.7	33.5	23.8	18.3
Visits								
≥3 ED	23.1	40.0	22.7	23.2	22.1	20.0	28.1	16.5
≥5 PCP	14.0	22.2	15.9	7.3	22.6	8.3	17.7	9.1
No outpatient visits	11.1	9.2	6.4	26.5	4.0	7.4	8.1	13.1
Home health care	18.6	38.4	19.8	33.6	15.4	12.6	9.9	10.5
Skilled nursing facility stay	9.7	20.0	7.3	23.4	5.9	4.5	4.5	6.3
Mental health visit	14.8	18.4	5.3	6.7	12.4	8.7	59.8	6.3

Abbreviations: COPS-2, Comorbidity Point Score, version 2; CVD, cardiovascular disease; ED, emergency department; LOH, likelihood of hospitalization; PCP, primary care physician.

^a^ Data are presented as the percentage of patients unless otherwise indicated.

### Patient Profiles

The data in Figure 1 and Table 1 were presented to the clinical stakeholders for review and discussion. The group agreed on the following final descriptive labels for these 7 complex patient profiles: highest acuity (highest inpatient and outpatient utilization with the most comorbid conditions), older patients with cardiovascular (older patients with a high prevalence of cardiovascular disease–related conditions and complications), frail elderly (oldest group with the highest 1-year mortality and the most frailty-related needs), chronic pain management (high outpatient utilization and mental health needs complicated by ongoing prescription of opioid-related drugs), active cancer treatment (intensive oncologic therapy with associated medical and pain management issues), psychiatric illness (severe mental illness complicated by low income, social needs, and pain management), and less clinically engaged (prevalent comorbidities but fewer visits).

Further examination of the clinical utilization and comorbidity patterns in conjunction with the patient clinical profiles also suggested specific strategies for how existing clinical care tools and programs could be tailored to meet the different complex medical and social needs for each patient profile. These optimal care strategies are summarized in Table 2.

**Table 2.** Key Defining Features and Suggested Management Strategies for the 7 Clinical Profiles of Medically Complex Patients

Profile	Key defining features	Suggested management strategies
Highest acuity	Patients with highest utilization (both inpatient and outpatient) with most comorbid conditions	Interdisciplinary care teams able to coordinate care (including transitions between care settings) and to address functional decline
Older patients with CVD	Older patients with high prevalence of CVD-related conditions and complications	CVD management with expanded focus to include addressing age- and SES-related barriers to health care
Frail elderly	Oldest group with highest 1-y mortality and most frailty-related needs	Geriatric care including coordination with hospice and use of advanced directives
Chronic pain management	High outpatient utilization and medical needs complicated by mental health needs	Coordinated pain management infrastructure (eg, opiate prescription guidelines, non–medication-based modalities)
Active cancer treatment	Intensive oncologic therapy with associated medical and pain management issues	Oncology care management and care coordination to address pain and other medical needs
Psychiatric illness	Severe mental illness complicated by low income, social needs, and pain management	Collaboration between traditional mental health services and care coordination and social work resources
Less clinically engaged	Prevalent comorbidities but fewer visits	Potential for improving care and preventing future complications through active outreach and engagement

Abbreviations: CVD, cardiovascular disease; SES, socioeconomic status.

### Comparison of LCA Results With GLRM and k-Means Clustering Results

**Figure 2.**
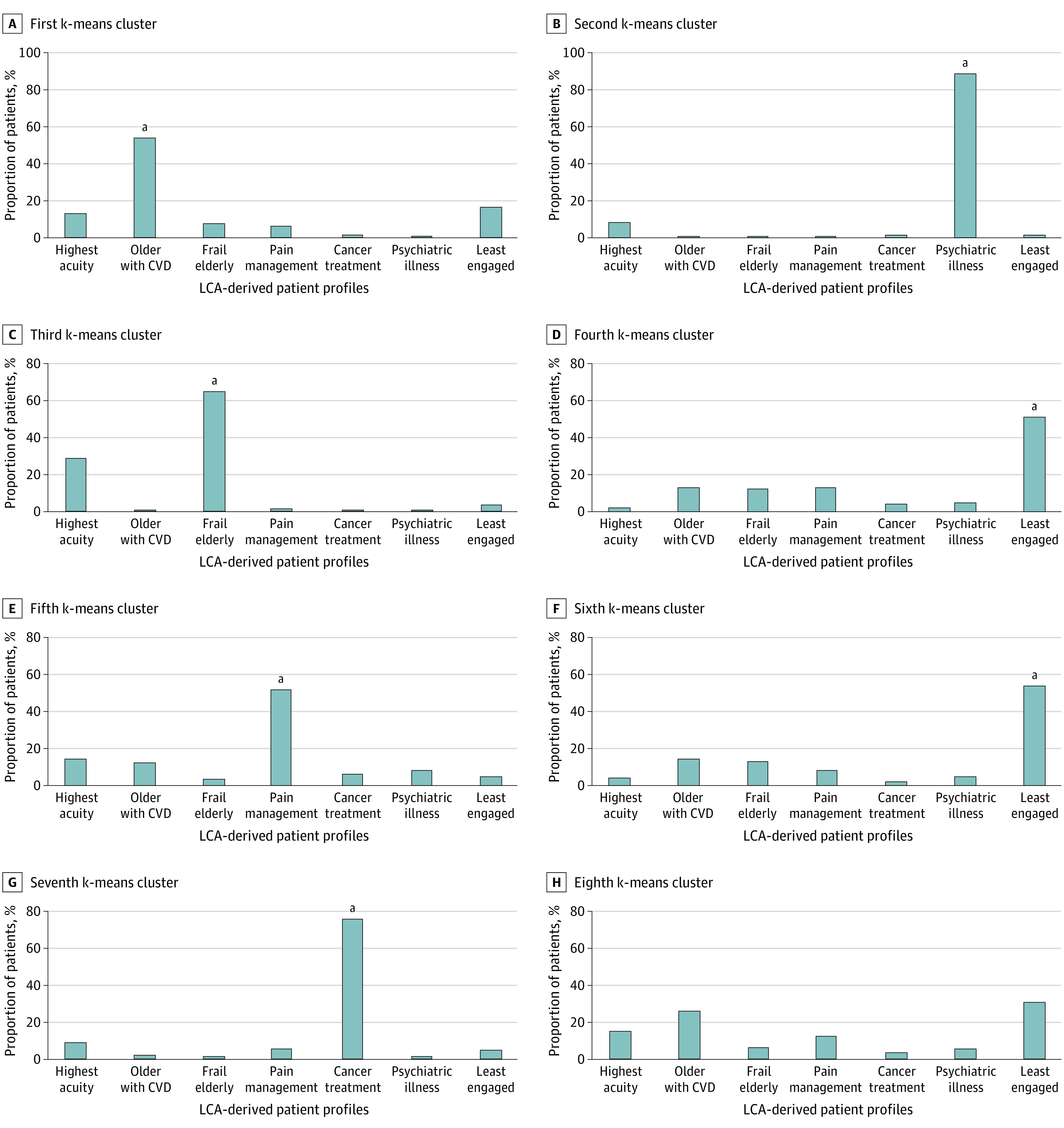
Comparison of k-Means Clustering With Latent Class Analysis (LCA) CVD indicates cardiovascular disease. ^a^Overlap between k-means and LCA clusters.

The GLRM and k-means clustering approach yielded an 8-class solution. We investigated the extent to which patients assigned to these 8 clusters matched the 7 profiles derived from the LCA. As shown in Figure 2, most patients in 7 of the 8 k-means clusters were primarily in a single LCA-derived patient profile. For example, 54% of patients in the second k-means cluster were in the older cardiovascular disease LCA cluster and 88% of patients in the first k-means cluster were in the psychiatric illness LCA cluster. The overlap was very high for 2 k-means clusters (>75% of patients in each k-means cluster patients were included in the active cancer treatment or psychiatric illness LCA-derived clinical profiles) and moderately high for 5 k-means clusters (50%-75% of patients in each cluster were in a single LCA-derived profile with <15% of patients in that cluster represented in another profile). The eighth k-means cluster (characterized primarily by obesity and insulin requirement) had patients represented in several different LCA groups with no one profile capturing more than 33% of the patients. Relative risk for 1-year outcomes by complex patient profile defined by k-means clusters were similar to the risks defined by LCA clusters (eTable 4 in the Supplement).

We noted that the highest acuity cluster from the LCA did not have a corresponding cluster in k-means clustering. When we investigated the overlap between grouping methods, we found that patients defined within the LCA highest acuity group were redistributed to the frail elderly (25%), older with cardiovascular disease (17%), pain management (17%), and the new, eighth k-means cluster (16%) (which we characterized as primarily patients with obesity and diabetes requiring insulin), suggesting that k-means clustering did not separate by acuity. Within the LCA less engaged profile, k-means clustering identified 2 sub-types: low engagement defined primarily by lack of online patient portal registration and low income (30% of the LCA less engaged group) and low prevalence of all variables, suggesting low acuity and medical stability (46% of the LCA less engaged group).

## Discussion

We identified 7 distinct patient profiles within the top 3% of medical complexity by linking multidimensional, clinically guided clustering methods to stakeholder interpretation. Some profiles were narrowly defined (eg, patients undergoing advanced cancer treatment, patients with severe mental illness), whereas others reflected distinct facets within the general complexity phenotype (eg, high acuity requiring substantial inpatient care, frail elderly, and older patients requiring advanced care for complications of cardiovascular disease). The remaining 2 profiles were defined by need (chronic pain management) or lack thereof (relatively limited health system contact despite high levels of calculated comorbidity and risk).

Our results provide empirical data that may inform conceptual models of complexity in adult populations and further support the diversity of high-need patients.^22,23^ One of the most prominent taxonomies, based on expert consensus by the National Academy of Medicines, created 6 categories of medically complex patients: children with complex needs, nonelderly disabled adults, frail elderly individuals, patients with major complex chronic conditions, patients with less severe but multiple chronic conditions, and patients with advancing illness. Such consensus-based models are a useful starting point but lack the empirical basis for redesigning care.^24^ Our approach combined data and consensus to better elucidate the heterogeneity within a narrower band of adult patients (top 3%) who are often indistinguishable using typical cost or diagnosis-based segmentation methods. By identifying the key variables that define different patient profiles, our study also offers data to guide efforts for operationalizing patient identification and to outline the types of different services and workforce competencies that may be required for complex care management (Table 2).

Each of these profiles suggested strategies for organizing care. Of note, although some profiles were labeled by a key distinction, such as undergoing chemotherapy, every patient in each profile also had multiple other chronic conditions. Consequently, care programs focused solely on supporting a single issue (eg, cancer care or mental illness) are not likely to fit the full range of needs in this medically complex patient population. Health care systems responsible for population care should implement strategies that go beyond directly supporting the immediate treatment needs to also address the impact of concurrent conditions.^25^ Several of the medically complex patient profiles identified in our study have been described in different settings and contexts. For example, chronic pain management is a well-recognized clinical challenge, likely even more so for patients with multiple chronic conditions. Our findings suggest that pain management is a concern for a large segment of the medically complex patient population that may require continued investment and well-designed care teams. Other research has described the unique needs of medically complex patients with cardiovascular disease^26^ and the importance of specific communication plans to address the needs of frail elderly adults with limited life expectancy.^27^ Several studies have shown the benefit of comprehensive care for frail elderly adults.^28^ Among the 1-year outcomes, hospice admission rates were nearly double among frail elderly adults compared with the high acuity group (17% vs 10%) despite similar mortality rates. This contrast suggests a need for program interventions that identify patients near the end of life so that they can be referred to appropriate care services.

In addition, some insight from the GLRM and k-means clustering suggests that less engaged patients, the largest group, could reflect 2 types of patients: those who are less engaged with care owing to barriers such as lack of access and those who appear to be medically complex but are actually medically stable. Knowing more about this group may help tailor outreach and potentially redirect resources to patients in other clusters most likely to benefit.

Our results have several implications for organizing care. Efforts to implement care strategies to address the needs of medically complex patients require innovation.^12,29^ Given the demonstrated heterogeneity within our narrow band of highly medically complex patients, our results suggest that a single care model is not likely to meet the needs of all complex patient subpopulations. Accountable care organizations responsible for patients with complex care needs can no longer solely rely on prior or predicted utilization or comorbidity summaries when identifying patients for care management. Efforts to tailor interventions may be guided by the needs suggested for each of the different profiles. However, creating 7 separate programs for this small but heterogeneous population segment may be impractical, and thus strategies should be developed that address shared needs across groups and that can implement tailored needs assessment to direct patients to specific resources. For example, some patients (eg, older patients with cardiovascular disease) might benefit from a care manager within primary care, whereas others (eg, patients receiving cancer treatment) might benefit from a comprehensive specialty care program. Future analytic work in this domain could involve creating algorithms to identify patients based on addressable needs, modeling to predict when patients might be on a trajectory toward one of these complex profiles, categorizing the major pathways to becoming a medically complex patient, and identifying preventive interventions that could aim to slow transition into these groups over time.

### Strengths and Limitations

This study has strengths. We analyzed highly dimensional clinical data for a large cohort of patients representing the most medically complex patients (top 3%) within a health care system. Other large studies in this area have been limited by reliance on administrative data for large general populations (leading to self-evident divisions between healthier and less healthy categories),^30,31,32^ and studies with greater depth of clinical data have been limited to smaller populations.^33,34,35^ Moreover, studies relying largely on diagnosis codes, while able to identify which medical conditions cooccur, may lack the ability to distinguish patients based on severity of illness, functional status, medication needs, or nonmedical factors that are associated with health outcomes and therefore are limited in their ability to suggest specific care innovations.^36,37,38,39^ Results from diagnosis code analyses are useful for identifying which conditions are enriched or cooccur in high-cost conditions (eg, end-stage renal disease, diabetes with multiple comorbidities, and acute on chronic illness) but are less helpful for designing care around patient-centered phenotypes (eg, frail elderly adults, chronic pain management).^40^ In addition, we linked our analytic results to clinical interpretation. Studies that apply automated approaches may return clusters of limited clinical interpretability (eg, creating groups distinguished by 1-year outcome risks that lack unifying clinical themes that could inform care redesign).^34^ Prior research has shown the value of asking clinicians to define patient complexity.^41,42,43,44^ This approach allowed us to extend the value of analytic clustering by developing specific suggestions for tailoring care needs within the medically complex patient population.

An important concern for all health care-related machine learning analyses is the potential for perpetuating disparities because data obtained as part of routine care can be inaccurate or incomplete in a way that may be biased toward different patient racial/ethnic groups.^45^ Although this concern is particularly an issue for prediction models used to assign health care resources to different patients, all such modeling should be cognizant of this problem.^46^ With these concerns in mind, we incorporated principles of distributive justice into our model design and evaluation to ensure robust results and to minimize biased data and overreliance on automation.^47^ These efforts included stakeholder collaboration to mediate the data through the clinical perspective to focus on outcomes that are meaningful to the target population; creating a data structure designed to avoid reinforcing health disparities by addressing nonrandom missingness (eg, specifying variables to represent lack of annual visits, screening, and health care contacts); optimizing completeness (KPNC includes out-of-system claims and has high-quality self-reported race/ethnicity coding); including utilization accumulated through nonvisit interactions to reduce bias against patients less likely to attend in-person visits (eg, telephone calls that often replace in-person visits for patients with transportation barriers)^48^; and model building that emphasized transparency and error assessment at all stages of variable creation and testing.^49^

This study also has limitations. Unlike established predictive modeling, there is currently no single gold standard for how to statistically validate data clustering results. To address this limitation, we examined validity within 3 domains: face validity (clusters corresponded to recognizable medically complex patient profiles by experts in the field), construct validity (2 methodologically unrelated clustering methods resulted in a qualitatively reassuring degree of overlap), and criterion validity (the different profiles had significantly different 1-year outcomes, demonstrating high correlation between profiles with external criteria). Another limitation was that our analysis was conducted within a single integrated care delivery system, which may limit generalizability. However, although the specific variables contributing to the clustering models may vary if replicated elsewhere, the corresponding patient clinical profiles developed by the clinical stakeholders were based on clinical experiences that are generalizable across care systems. In addition, although there are advantages to clinically guided model development, automated machine learning (ie, without clinical guidance) can potentially generate novel insights that are not readily apparent. However, such insights may not be actionable without broader clinical context.

## Conclusions

The findings suggest that a single care model may not meet the needs of adults with high comorbidity and care utilization. Highly medically complex patient populations may be categorized into distinct patient profiles that are amenable to varying strategies for resource allocation and coordinated care interventions.

## Notes

Supplement. **eTable 1.** Data Description and Variable Extraction Strategy by Health Information Domain **eTable 2.** Overall Observed Prevalence and Predicted Prevalence of Variable Endorsement Across Classes **eTable 3.** Relative Risk for 1-year Outcomes by Complex Patient Profile, LCA Clusters **eTable 4.** Relative Risk for 1-year Outcomes by Complex Patient Profile, K-means clusters
