# Supplementary material for: Use of Latent Class Analysis and k-Means Clustering to Identify Complex Patient Profiles
Source: JAMA Netw Open. 2020 Dec 11;3(12):e2029068. doi: 10.1001/jamanetworkopen.2020.29068 (PMC7733156; doi:10.1001/jamanetworkopen.2020.29068)
Supplement: Supplement. — eTable 1. Data Description and Variable Extraction Strategy by Health Information Domain eTable 2. Overall Observed Prevalence and Predicted Prevalence of Variable Endorsement Across Classes eTable 3. Relative Risk for 1-year Outcomes by Complex Patient Profile, LCA Clusters eTable 4. Relative Risk for 1-year Outcomes by Complex Patient Profile, K-means clusters [file jamanetwopen-e2029068-s001.pdf]

Supplemental Online Content

Grant RW, McCloskey J, Hatfield M, et al. Use of latent class analysis and k-means clustering to identify complex patient profiles. *JAMA Netw Open.* 2020;3(12):e2029068. doi:10.1001/jamanetworkopen.2020.29068

eTable 1. Data Description and Variable Extraction Strategy by Health Information Domain

eTable 2. Overall Observed Prevalence and Predicted Prevalence of Variable Endorsement Across Classes

eTable 3. Relative Risk for 1-year Outcomes by Complex Patient Profile, LCA Clusters

eTable 4. Relative Risk for 1-year Outcomes by Complex Patient Profile, K-means clusters

This supplemental material has been provided by the authors to give readers additional information about their work.

© 2020 Grant RW et al. *JAMA Network Open.*

MOMO Feature Extraction Summary

eTable 1. Data Description and Variable Extraction Strategy by Health Information Domain
All model variables were generated from extensive electronic data residing in multiple data systems within our electronic health record. Our aim was to create variables that represented the breadth of health information domains consistently available for all patients treated by the health system. Each data domain had a unique structure and relationship between data entries or records. As such, the extraction strategy was adapted to suit the properties of each domain. For all domains, we extracted patient data for one year prior to the index date of July 15, 2018. This Table provides detail to the variable creation process used for clustering listed in eTable 2.

Domain	Data Sources and Properties	Variable Extraction Strategy
Communications	Phone calls documented by clinicians and staff; registration/log-in information and email messages captured from the online patient portal	Restricted to messages with patient's PCP and incoming calls that were not automated (e.g. excluded pacemaker initiated); grouped by patient initiation to create count variables
Disease Registries	End-stage renal disease captured from population health management tool updated monthly	Latest entry used
Durable Medical Equipment (DME)	All DME orders with numeric codes grouped by equipment type	Clinically guided grouping and selection of relevant DME based on code description
Health Behaviors/ Depression	Patient responses to questionnaires administered and vital signs collected during clinical encounters	Latest entry used
Lab Results	Lab results flagged as abnormal within electronic systems	Clinically guided selection and grouping of relevant labs to create indicator variables
Medications	All filled prescriptions, each linked to a Generic Product Identifier (GPI) hierarchical classification system code	Grouped by the first four characters of GPI code; indicator variables created for each medication type
Membership	Health plan administrative data related to coverage periods and plan type	Membership records merged to define continuous membership periods; indicator variables created for each membership type
Procedures	All procedures captured in electronic health record and claims systems.	Clinically guided grouping using procedure code taxonomy and code description; indicator variables created for each procedure group
Sociodemographics	Emergency contact information and need for an interpreter data were available in real-time from the electronic health record; Neighborhood Deprivation Index Score calculated from 2016 American Community Survey data by census tract and linked via geocoded patient addresses; medical	Latest entry used except for medical financial assistance (indicator variable created for any financial assistance application or coverage in the prior year)

© 2020 Grant RW et al. *JAMA Network Open.*

	financial assistance data stored by unique application and coverage period.	
Utilization	All patient visits and facility admissions captured in the electronic health record and claims systems; each encounter had an associated a type and subtype, generated primarily from location of service; PCP assignment data from an administrative source linked to visits using a unique provider ID.	Grouped primarily by encounter type with subgrouping by department, provider specialty and provider type when greater specificity was needed; count variables created for each clinically selected utilization group; visit no shows grouped by chemical dependency/psychiatry department or not, due to higher frequency (weekly) of behavioral health visits.

© 2020 Grant RW et al. *JAMA Network Open.*

eTable 2. Overall Observed Prevalence and Predicted Prevalence of Variable Endorsement Across Classes

Variables are sorted by class count (descending greater or equal to 55% then ascending less than or equal to 10%) then descending observed prevalence. Box colors indicate variable prevalence category. Variables boxed in **bold** had the largest variation in prevalence across classes.

	Overall Observed Prevalence	Class count		Highest Acuity	Older w CVD	Frail Elderly	Chronic Pain	Active Cancer	Psych Illness	Less Engaged
		≥ 55%	≤ 10%							
Number of patients	***104,869***			***10,360***	***17,524***	***13,786***	***13,557***	***7,967***	***12,545***	***29,130***
	%			%	%	%	%	%	%	%
Tobacco use: Any use in lifetime	53.2	4	0	59.0	57.9	49.0	55.2	46.5	55.4	50.3
**Medication: Opioid Agonists**	51.5	4	0	76.8	39.4	41.4	81.1	70.6	56.2	33.5
Exercise: 0 minutes per week	57.1	3	0	69.0	59.9	71.6	54.8	42.7	50.8	52.0
**Utilization: 1+ Hospital admission**	41.1	3	1	93.3	45.9	75.3	28.1	55.4	32.5	9.5
**Medication: Antidepressants**	37.9	2	0	55.6	19.9	38.5	52.2	28.5	76.6	21.4
**Labs: Stage 3-4 kidney disease**	35.4	2	0	55.2	56.0	47.8	24.2	32.3	17.6	23.9
**Medication: Diuretics - Loop**	31.8	2	0	66.7	73.2	28.7	17.6	16.8	12.9	14.9
**Utilization: 7+ Outpatient departments visited**	28.3	2	1	63.7	24.9	10.7	57.7	44.5	37.7	3.8
**Utilization: 1+ Home health visit**	25.6	2	2	74.5	17.0	76.7	17.7	16.1	8.9	2.8
**Labs: BNP– high**	25.1	2	4	68.1	61.9	34.3	7.0	9.3	6.1	4.2
**Medication: Anticonvulsants - Not Benzodiazepines**	28.7	1	0	48.5	15.5	23.8	46.0	22.6	55.2	14.2
**Labs: liver enzymes – high**	26.5	1	0	53.8	22.7	28.3	15.4	62.1	28.2	13.0
**Medication: Glucocorticoids**	25.7	1	0	39.0	24.9	12.2	38.0	55.2	24.0	14.6
**Labs: Albumin – low**	23.6	1	2	66.7	24.2	44.4	8.4	46.5	10.0	4.6

© 2020 Grant RW et al. *JAMA Network Open.*

**Labs: Platelets - low**	21.8	1	2	45.7	26.2	28.4	7.7	56.5	12.3	8.7
**Home equipment or wheelchair**	20.3	1	3	51.6	8.1	59.2	14.3	13.2	9.4	7.4
**Utilization: 1+ Mental health visit**	16.7	1	5	25.9	3.7	7.8	6.7	7.7	86.5	2.6
**Procedures: Psychiatric care**	11.3	1	5	14.7	1.0	1.9	0.5	4.3	73.9	0.7
**Procedures: Cancer (chemo, radiation, surgery)**	7.7	1	6	9.1	1.4	1.5	4.4	66.8	2.7	1.3
Neighborhood deprivation index: Quartile 3 or 4	47.9	0	0	50.8	44.6	42.8	46.2	42.7	53.1	51.1
Body Mass Index 30+	38.1	0	0	39.9	45.2	21.4	47.7	24.2	44.9	37.3
Utilization: 3+ ED visits not admitted to hospital	24.2	0	0	38.1	13.8	21.5	31.4	15.6	40.0	19.1
Utilization: 7+ Patient Messages to PCP	24.0	0	1	35.9	20.8	19.1	45.5	25.1	33.1	9.9
Medication: Anticoagulants - Not Heparin	21.5	0	1	38.4	49.4	19.4	15.9	10.1	7.6	11.5
Utilization: 5+ Primary care visits	20.7	0	1	43.5	23.1	12.2	35.7	10.8	26.8	8.3
Procedures: Physical therapy	18.0	0	1	26.6	10.1	14.3	46.2	13.4	22.7	7.5
Procedures: Cardiac stress test	18.6	0	2	31.6	40.6	4.1	19.9	21.2	12.5	8.8
DME: Mobility walking	16.0	0	2	42.4	10.0	33.2	17.5	15.3	10.1	4.3
Utilization: 2+ Social work visit	20.5	0	3	45.4	6.6	32.7	6.9	29.1	51.8	4.9
Utilization: 1+ Skilled nursing facility stay	10.9	0	5	35.3	0.7	46.1	1.8	2.0	2.5	2.1
PHQ9 Depression score 10+	9.7	0	5	14.8	2.4	3.7	7.4	3.7	44.8	2.9
Labs: Neutrophils - low	7.8	0	5	12.6	2.8	4.4	2.5	49.6	6.3	2.6
Utilization: Not registered on online patient portal	24.9	0	0	23.3	23.7	32.0	14.1	13.6	20.5	33.0
Medication: Insulins	18.2	0	0	35.3	24.9	11.6	17.2	11.5	13.2	15.7

© 2020 Grant RW et al. *JAMA Network Open.*

Medications: <80% Possession ratio	17.1	0	0	27.2	17.3	20.2	14.5	12.6	14.3	15.5
Medical financial assistance application or coverage	14.5	0	1	24.0	14.9	13.3	14.6	20.2	15.1	9.6
No in-state emergency contact on file	17.9	0	2	7.2	9.9	14.0	13.5	10.8	30.2	27.2
PCP assignment: 1+ Patient initiated switch	13.1	0	2	18.2	9.9	14.6	15.7	11.2	19.2	9.2

Medication: Antianxiety Agents – Benzodiazepines	15.4	0	3	19.2	5.1	9.6	21.6	28.9	37.4	6.9
Medication: Anti asthmatic - Steroid Inhalants	14.3	0	3	21.3	18.1	8.3	21.1	8.7	16.3	9.8
Labs: Eosinophils - high	11.8	0	3	27.4	10.4	12.7	8.0	32.0	7.3	4.9
Medication: Antipsychotics or Antimanic Agents	11.8	0	3	13.3	0.9	15.4	2.9	34.4	36.3	3.4
Medication: Musculoskeletal Therapy Agents	12.2	0	4	15.7	4.2	4.1	29.8	8.5	25.1	6.9
DME: Oxygen	9.9	0	4	25.2	14.4	13.4	7.1	4.9	3.7	5.3
Medication: Gout Agents	9.6	0	4	16.9	18.8	6.0	6.4	10.7	3.5	6.8
End-stage renal disease (on registry, lab or procedure)	8.0	0	4	23.0	12.1	4.4	2.4	10.4	1.8	6.7
Utilization: No office or telephone visits	18.6	0	5	4.2	9.5	36.0	4.0	1.8	8.1	36.9
Medication: Antianginal Agents	11.8	0	5	27.8	29.5	7.3	7.3	2.2	4.3	5.7
Medication: Platelet Aggregation Inhibitors	9.7	0	5	19.7	18.4	9.3	8.0	2.4	4.0	6.4
Medications: High risk medications	9.6	0	5	16.7	6.5	8.0	19.5	9.9	8.3	5.5

© 2020 Grant RW et al. *JAMA Network Open.*

Procedures: Speech assessment and treatment	9.0	0	5	28.2	2.9	31.4	4.2	4.9	4.2	0.8
Procedures: Hearing assessment and treatment	7.7	0	5	10.8	8.7	5.8	12.3	8.0	6.8	5.0
Utilization: No recent login to online patient portal	7.6	0	5	7.1	6.3	12.1	4.2	3.3	5.7	10.1
Procedures: Lung function assessment	6.3	0	5	11.4	12.0	0.8	9.2	9.4	4.5	2.1
Labs: INR/PTT - high	5.3	0	5	20.1	10.1	5.0	1.5	5.9	1.2	0.5
Medication: Antiarrhythmics	4.8	0	5	11.5	15.2	2.0	2.1	1.1	1.2	1.1
Utilization: 7+ Patient calls	4.5	0	5	11.2	2.6	0.6	5.6	11.8	9.3	0.6
Procedures: Cardiovascular (major surgery, catheterization)	3.5	0	5	11.8	11.1	0.4	1.6	1.2	1.0	0.2

Any Medicaid membership during prior year	8.4	0	6	9.9	3.6	4.6	8.9	6.1	20.2	7.7
Labs: HbA1c- high (> 9%)	8.3	0	6	13.7	9.4	5.0	7.1	4.4	8.2	8.9
Medication: Antineoplastic or Adjunctive Therapies	7.0	0	6	8.3	3.7	4.1	6.2	36.8	3.3	3.7
Medication: Urinary Antispasmodics	6.1	0	6	9.1	3.5	8.0	11.2	4.0	6.5	3.8
Procedures: Cardiac arrhythmia assessment and treatment	5.6	0	6	9.5	17.0	3.5	3.0	1.0	1.8	2.4
Medication: Bupropion	4.9	0	6	6.6	1.3	1.8	6.9	2.5	18.1	1.9
Labs: Lipase - high	4.6	0	6	11.8	2.7	4.8	2.4	8.7	5.4	2.7
Utilization: 4+ No shows to primary care visits	4.0	0	6	11.5	2.2	1.7	4.6	2.3	8.6	1.6
Medication: Smoking Deterrents	4.2	0	6	6.3	2.7	2.2	4.4	3.2	11.2	2.3

© 2020 Grant RW et al. *JAMA Network Open.*

Labs: Potassium - critical low	3.9	0	6	17.2	4.0	4.6	0.7	5.2	1.5	0.8
Utilization: 1+ Chemical dependency visits	3.6	0	6	3.4	0.2	0.1	0.5	2.2	23.4	0.8
Medication: Antidementia	3.3	0	6	2.4	1.1	10.7	1.8	0.8	2.8	3.0
Procedures: Echocardiography	3.1	0	6	12.3	5.7	3.3	0.6	1.8	1.3	0.3
Medication: Immunomodulators or Suppressors	3.0	0	6	6.0	2.6	0.3	1.9	14.7	1.2	1.4
Medication: Benzodiazepines	2.6	0	6	3.4	0.6	1.0	2.9	1.4	11.4	0.7
Procedures: Liver/GI procedures	2.4	0	6	6.1	1.4	1.7	0.7	12.9	1.3	0.5
Labs: Potassium - critical high	2.1	0	6	10.8	2.5	1.5	0.3	1.8	0.3	0.6
Utilization: 4+ No shows to behavioral health visits	2.0	0	6	1.3	0.0	0.0	0.0	0.1	15.5	0.0
Medication: Growth Factors	1.9	0	6	3.6	0.8	0.2	0.0	16.9	0.1	0.3
Medication: Hypnotics, Sedatives, Sleep Disorder Agents	4.8	0	7	7.4	2.4	4.4	7.2	8.4	9.4	1.6
Medication: Pulmonary Hypertension	4.6	0	7	4.3	4.8	0.7	6.2	5.6	5.9	4.7
Procedures: Sleep assessment	4.5	0	7	7.8	6.3	1.9	6.8	1.8	6.9	2.0
Medication: Parkinson's Agents	3.9	0	7	6.2	1.6	6.2	5.8	1.1	7.8	1.7
Social: Needs Interpreter	3.7	0	7	3.1	3.2	4.2	2.2	3.7	2.2	5.3
Procedures: Osteoporosis assessment	3.5	0	7	5.0	3.1	2.7	6.2	4.5	2.6	2.4
Labs: Influenza - positive	3.4	0	7	6.6	3.5	6.0	2.4	2.8	2.1	2.2
Utilization: Less than 50% of 3+ primary care visits with PCP	3.2	0	7	3.4	2.7	2.0	4.5	3.1	4.7	2.6
Alcohol use: Exceeds daily or weekly limit	3.0	0	7	1.2	3.1	1.8	2.8	3.1	4.9	3.4
Medication: Migraine Products	2.2	0	7	1.6	0.3	0.2	5.0	1.5	8.1	1.0

© 2020 Grant RW et al. *JAMA Network Open.*

Procedures: Major joint replacement	2.1	0	7	4.4	0.1	5.7	5.8	0.4	1.1	0.0
Medication: Antivirals	2.0	0	7	2.9	0.7	0.1	1.0	8.6	2.9	1.5
Procedure: Low ejection fraction (< 30%)	2.0	0	7	4.5	7.9	0.6	0.2	0.3	0.3	0.3
Utilization: Hospice (on registry or received care)	2.0	0	7	3.2	0.4	8.8	0.0	3.8	0.3	0.5
Labs: PSA (prostate-specific antigen) – high	1.8	0	7	1.6	2.1	1.2	1.4	5.6	0.7	1.6
Labs: LDH (lactate dehydrogenase) - high	1.3	0	7	4.8	0.7	0.5	0.0	7.0	0.3	0.1
Medications: Opioids	1.3	0	7	2.0	0.2	0.5	2.8	0.3	3.9	0.5
Procedures: Nerve studies	1.3	0	7	2.4	0.5	0.3	4.1	0.8	2.3	0.3
Medications: Opioid antagonists/antidotes	1.1	0	7	0.8	0.0	0.1	0.6	0.2	7.0	0.2
Procedures: Bronchoscopy	1.0	0	7	2.5	0.5	0.3	1.3	5.0	0.3	0.1

© 2020 Grant RW et al. *JAMA Network Open.*

eTable 3. Relative Risk for 1-year Outcomes by Complex Patient Profile, LCA Clusters

1-Year Outcome	Highest Acuity	Older w CVD	Frail Elderly	Chronic Pain	Active Cancer	Psych Illness	Less Engaged (referent)
Died	3.4 (3.2, 3.6)	1.6 (1.5, 1.8)	3.9 (3.7, 4.1)	0.6 (0.6, 0.7)	3.6 (3.4, 3.8)	0.5 (0.4, 0.6)	1
Admitted to hospice	3.2 (2.9, 3.5)	1.2 (1.1, 1.2)	5.3 (4.9, 5.7)	0.6 (0.5, 0.7)	4.9 (4.5, 5.3)	0.5 (0.4, 0.6)	1
Admitted to hospital	2.8 (2.7, 2.9)	1.8 (1.8, 1.8)	1.9 (1.8, 2.0)	1.2 (1.19, 1.3)	1.8 (1.76, 1.9)	1.3 (1.25, 1.35)	1
3+ ED visits	2.4 (2.3, 2.5)	1.4 (1.3, 1.4)	1.4 (1.35, 1.46)	1.3 (1.28, 1.4)	1.2 (1.15, 1.3)	1.7 (1.6, 1.8)	1
5+ PCP visits	2.4 (2.3, 2.6)	1.8 (1.7, 1.8)	0.8 (0.7, 0.9)	2.5 (2.4, 2.6)	0.9 (0.8, 1.0)	2.0 (1.9, 2.1)	1
No visits	0.7 (0.6, 0.8)	0.5 (0.46, 0.53)	2.0 (1.95, 2.1)	0.3 (0.28, 0.33)	0.6 (0.5, 0.6)	0.6 (0.6, 0.7)	1
Home health visit	3.6 (3.5, 3.8)	1.8 (1.8, 2.0)	3.2 (3.1, 3.3)	1.5 (1.4, 1.5)	1.2 (1.1, 1.3)	0.9 (0.9, 1.00)	1
SNF stay	3.2 (2.0, 3.4)	1.2 (1.2, 1.2)	3.7 (3.5, 3.9)	0.9 (0.9, 1.0)	0.7 (0.6, 0.8)	0.7 (0.6, 0.8)	1
Mental health visit	2.9 (2.7, 3.1)	0.8 (0.8, 0.9)	1.1 (1.0, 1.2)	2.0 (1.9, 2.1)	1.4 (1.3, 1.5)	9.5 (9.0, 9.9)	1

Numbers are relative risks and 95% confidence intervals, with the Less Engaged profile as the referent. ED = Emergency Department, PCP = Primary Care Provider, SNF = Skilled Nursing Facility

© 2020 Grant RW et al. *JAMA Network Open.*

eTable 4. Relative Risk for 1-year Outcomes by Complex Patient Profile, K-means clusters

**1-Year Outcome**	Skilled Nursing and High Acuity	Less Engaged Low Income	Pain Management	Complex Diabetes	Cancer Treatment	Psychiatric Illness	Complex with CVD	Less Engaged Stable (Referent)
Died	2.5 (2.4, 2.7)	1.07 (1.01, 1.14)	0.62 (0.57, 0.67)	0.86 (0.80, 0.92)	2.8 (2.7, 3.0)	0.44 (0.39, 0.49)	1.25 (1.17, 1.33)	1
Admitted to hospice	2.3 (2.1, 2.4)	0.83 (0.77, 0.90)	0.47 (0.42, 0.53)	0.50 (0.45, 0.56)	3.1 (2.9, 3.3)	0.32 (0.27, 0.37)	0.84 (0.77, 0.92)	1
Admitted to hospital	1.8 (1.77, 1.89)	1.18 (1.14, 1.22)	1.23 (1.18, 1.27)	1.47 (1.42, 1.52)	1.52 (1.46, 1.59)	1.15 (1.10, 1.20)	1.45 (1.40, 1.50)	1
3+ ED visits	1.48 (1.42, 1.55)	1.35 (1.30, 1.40)	1.30 (1.25, 1.36)	1.45 (1.39, 1.51)	1.12 (1.06, 1.18)	1.54 (1.48, 1.60)	1.28 (1.23, 1.33)	1
5+ PCP visits	0.92 (0.86, 0.99)	1.32 (1.25, 1.39)	2.28 (2.17, 2.39)	1.51 (1.42, 1.59)	0.85 (0.78, 0.92)	1.71 (1.62, 1.81)	1.65 (1.56, 1.74)	1
No visits	2.0 (1.9, 2.1)	1.12 (1.07, 1.18)	0.28 (0.26, 0.31)	0.65 (0.61, 0.70)	0.56 (0.51, 0.61)	0.65 (0.61, 0.70)	0.59 (0.56, 0.64)	1
Home health visit	2.54 (2.44, 2.64)	1.11 (1.06, 1.16)	1.21 (1.16, 1.27)	1.31 (1.25, 1.37)	1.02 (0.96, 1.09)	0.74 (0.69, 0.79)	1.53 (1.47, 1.60)	1
SNF stay	6.40 (6.05, 6.77)	1.62 (1.52, 1.74)	1.11 (1.02, 1.20)	1.58 (1.46, 1.71)	1.00 (0.90, 1.12)	0.91 (0.83, 1.01)	1.54 (1.43, 1.66)	1
Mental health visit	1.05 (0.98, 1.13)	0.94 (0.88, 1.00)	1.75 (1.65, 1.84)	1.25 (1.17, 1.33)	0.97 (0.89, 1.05)	6.27 (6.02, 6.53)	0.72 (0.67, 0.78)	1

Numbers are relative risks and 95% confidence intervals, with the Less Engaged, Stable profile as the referent. ED = Emergency Department, PCP = Primary Care Provider

© 2020 Grant RW et al. *JAMA Network Open.*
